# Probability of twin formation on self-catalyzed GaAs nanowires on Si substrate

**DOI:** 10.1186/1556-276X-7-558

**Published:** 2012-10-08

**Authors:** Masahito Yamaguchi, Ji-Hyun Paek, Hiroshi Amano

**Affiliations:** 1 Department of Electrical Engineering and Computer Science, Nagoya University, C3-1 Furo-cho, Chikusa-ku, Nagoya, 464-8603, Japan

**Keywords:** GaAs nanowire, Molecular beam epitaxy, Vapor-liquid-solid, Twin boundary, Supersaturation, Wurtzite, zinc blende

## Abstract

We attempted to control the incorporation of twin boundaries in self-catalyzed GaAs nanowires (NWs). Self-catalyzed GaAs NWs were grown on a Si substrate under various arsenic pressures using molecular beam epitaxy and the vapor-liquid-solid method. When the arsenic flux is low, wurtzite structures are dominant in the GaAs NWs. On the other hand, zinc blende structures become dominant as the arsenic flux rises. We discussed this phenomenon on the basis of thermodynamics and examined the probability of twin-boundary formation in detail.

## Background

III-V compound semiconductor nanowires (NWs) have been attracting significant attention as fundamental structures of novel optical and electronic devices. Especially, vapor-liquid-solid (VLS) NWs grown on Si substrates have been investigated for optoelectronic integrated circuits [1,2].

Previously, we succeeded in growing self-catalyzed GaAs NWs on Si substrates using molecular beam epitaxy (MBE)-VLS method which is a combination of MBE and VLS method [3]. However, twin boundaries formed in the NWs during growth. The occurrence of twin boundaries is controlled by various methods such as control of supersaturation, growth temperature, and diameter [4-8]. To control the twin boundaries, we focused on the pressure of arsenic because self-catalyzed GaAs NW growth was nearly independent of Ga pressure, and arsenic flux plays an important role in the growth mechanism [3]. In addition, arsenic solubility in Ga solution is very low [9]. This means that the degree of supersaturation depends on arsenic pressure only.

## Methods

Self-catalyzed GaAs NWs were grown on a (111)Si substrate by MBE-VLS method. The growth temperature was 580°C. When the arsenic flux varied from 5.0×10^−6^ to 1.9×10^−5^ Torr, the diameter and the length of the obtained GaAs NWs varied from 90 to 30 nm and 0.5 to 3.5 μm, respectively. We have previously discussed the tendency of the diameter and length to depend on the arsenic flux [3]. The obtained NWs were observed by transmission electron microscopy (TEM).

## Results and discussion

**Figure 1:**
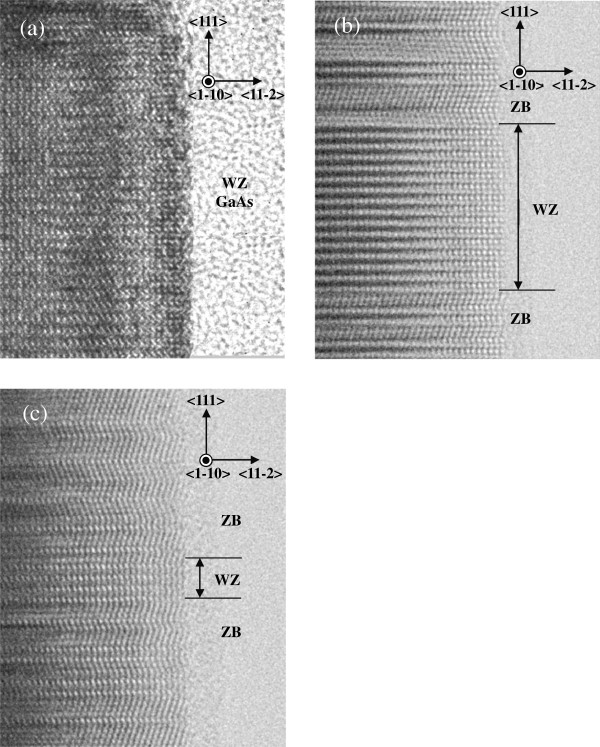
**TEM images of NWs grown under various arsenic fluxes.** (**a**) 5.0 × 10^−6^, (**b**) 7.0 × 10^−6^, and (**c**) 1.9 × 10^−5^ Torr.

TEM images of GaAs NWs are shown in Figure 1. In the case of low arsenic flux (5.0 × 10^−6^ Torr), wurtzite (WZ) structures are dominant as shown in Figure 1a. On the other hand, when the arsenic flux is high, Figure 1c shows that segments between the twin boundaries become large, and zinc blende (ZB) structures are dominant. The segment size follows the time between successive twin-crystal nucleation events. Furthermore, distribution of the segment sizes is exponential from a stochastic point of view [10].

**Figure 2:**
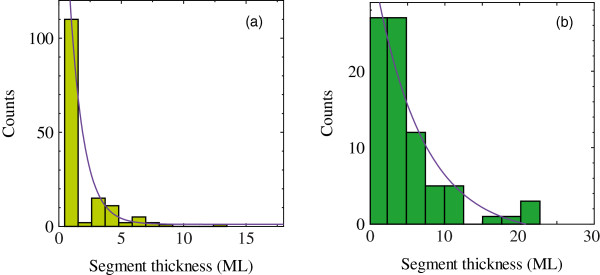
**Histogram and distribution of segment size obtained from Figure**1**.** Arsenic fluxes are (**a**) 7.0 × 10^−6^ and (**b**) 1.9 × 10^−5^ Torr. Solid lines are exponential fitting curves.

Figure 2 shows the distribution histogram of the segment sizes obtained from Figure 1. We assume a segment size of *x* and fitted the histogram with an exponential curve of the form exp (^−^*x*/*a*) to estimate the expectation value of segment α. The estimated expectation values were 1.1 and 6.5 monolayers at arsenic fluxes of 7.0 × 10^−6^ and 1.9 × 10^−5^ Torr, respectively. The reciprocal of the expectation value α^−1^ is equivalent to the probability of occurrence of twin-crystal nucleation. The probabilities were approximately 90% (7.0 × 10^−6^ Torr) and 15% (1.9 × 10^−5^ Torr). The dependence of the probability on the arsenic flux is in good agreement with [11] and [12].

To understand these phenomena, we calculate the degree of supersaturation and estimate the probability of twin-boundary formation, following the procedure presented by Glas [13]. The degree of supersaturation Δμ is as follows:

(1) $$\Delta \mu =\mu_{\text{Ga}}^{\text{L}}+\mu_{\text{As}}^{\text{L}}-2\mu_{\text{GaAs}}$$

where *μ*_GaAs_ is the half chemical potential of GaAs crystal nucleation; *μ*_Ga_^L^ and *μ*_As_^L^ are the chemical potentials of liquid gallium and liquid arsenic, respectively. We assume that the arsenic adatom was liquid arsenic existing on the gallium droplet. However, *μ*_Ga_^L^ and *μ*_As_^L^ include an interaction between gallium and arsenic in pure gallium and arsenic chemical potentials *μ*_Ga_^pL^ and *μ*_As_^pL^, respectively. Therefore, *μ*_*X*_^L^ (*X* = Ga or As) is given by

(2) $$\mu_{X}^{\text{L}}=\mu_{X}^{\text{pL}}+RT\;\text{ln}\left(a_{X}^{\text{L}}\right)$$

where *R* is the gas constant, *T* is the growth temperature, and $a\frac{\text{L}}{X}$ is the activity of *X* in the liquid phase. At *T*_0_ = 298.15 K, when we adopt an enthalpy per mol in the *X*, the solid phase of *h*_*X*,0_^pS^, the degree of supersaturation Δ*μ* is given by

(3) $$\Delta \mu =RT\;\text{ln}\left(a_{\text{Ga}}^{\text{L}}\right)+RT\;\text{ln}\left(a_{\text{As}}^{\text{L}}\right)+\left(\mu_{\text{Ga}}^{\text{L}}-h_{\text{Ga,}0}^{\text{pS}}\right)+\left(\mu_{\text{As}}^{\text{pL}}-h_{\text{As,}0}^{\text{ps}}\right)-2\left(\mu_{\text{GaAs}}-0.5h_{\text{Ga,}0}^{\text{pS}}-0.5h_{\text{As,}0}^{\text{pS}}\right)\text{.}$$

All terms except the enthalpies depend on the growth temperature. The third, fourth, and fifth terms are the differences between the chemical potential and enthalpy of the pure gallium droplet, arsenic droplet, and GaAs crystal. Their values are tabulated in the paper of Ansara et al. [14]. The first and second terms are concerned with interactions in the alloy. They are denoted by the atomic concentration *c*_*X*_ and the interaction parameter for gallium and arsenic *ω*_Ga,As_ as follows:

(4) $$RT\ln\left(a_{\text{Ga}\left(\text{As}\right)}^{\text{L}}\right)=RT\ln\left(c_{\text{Ga}\left(\text{As}\right)}\right)+c_{\text{As}\left(\text{Ga}\right)}^{2}\omega_{\text{Ga},\text{As}}=RT\ln\left(c_{\text{Ga}\left(\text{As}\right)}\right)+\frac{c_{\text{As}\left(\text{Ga}\right)}^{2}V_{\text{Ga}}V_{\text{As}}}{c_{\text{Ga}}V_{\text{Ga}}+c_{\text{As}}V_{\text{As}}}\left[{\left(\delta_{\text{Ga}}-\delta_{\text{As}}\right)}^{2}-\frac{1.256\times 10^{5}{\left(\chi_{\text{Ga}}-\chi_{\text{As}}\right)}^{2}}{\sqrt{V_{\text{Ga}}V_{\text{As}}}}\right]$$

where *V*_Ga(As)_, *χ*_Ga(As)_, and *δ*_Ga(As)_ are the molar volume in the liquid phase, Pauling electronegativity, and Hildebrand solubility parameter, respectively. From these equations, we can obtain the relational expression for the supersaturation dependence on growth temperature and arsenic concentration in the gallium droplet.

**Figure 3:**
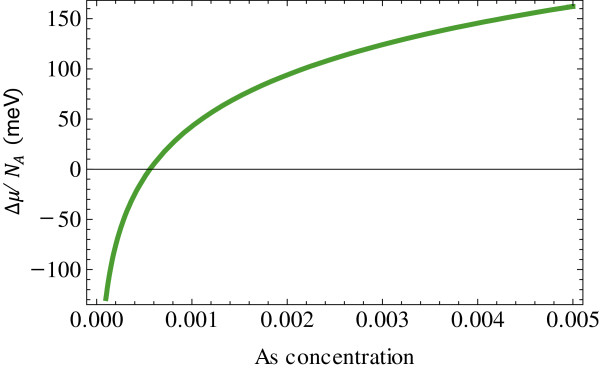
Supersaturation as a function of arsenic concentration in gallium droplet at 580°C.

Figure 3 shows the supersaturation as a function of the arsenic concentration in the gallium droplet at 580°C. In this calculation, the supersaturation per atom Δ*μ*/*N*_A_, where *N*_A_ is Avogadro's number, is adopted. In Figure 3, the equilibrium arsenic concentration is approximately 0.06% at Δ*μ*/*N*_A_ = 0. This value is in fair agreement with the arsenic concentration of approximately 0.1% in the gallium droplet at 580°C [9]. Therefore, when the arsenic concentration is positive because of an oversupply of arsenic atoms greater than 0.06%, GaAs VLS growth may occur.

**Figure 4:**
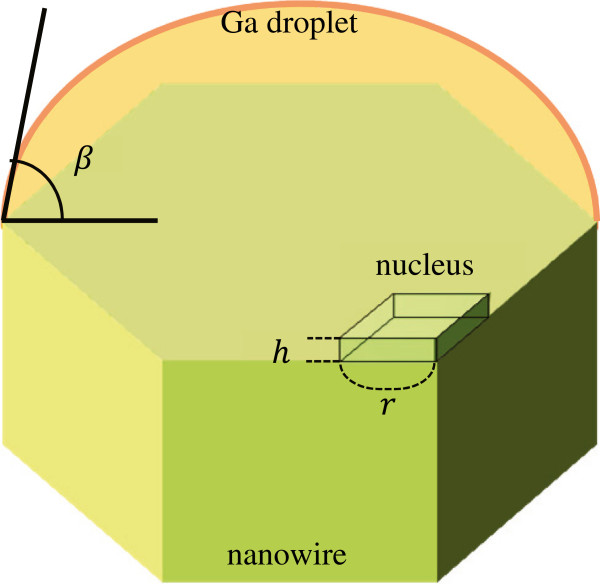
Growth model adopted in the calculation.

From supersaturation Δ*μ*/*N*_A_, we can obtain the probability of twin-boundary formation. When arsenic adatoms on the gallium droplet surface diffuse to the three-phase boundary of vapor, liquid, and solid phases, we assumed that the GaAs crystal nucleus would be formed into a rhombus shape with side length *r* at the vertex of the nanowire top surface. Figure 4 shows the growth model. When the ZB and WZ structures form, the amount of Gibbs free energy change in this growth system is given by

(5) $$\Delta G_{\text{ZB}\left(\text{WZ}\right)}=-\frac{\sqrt{3r^{2}h\Delta \mu }}{2\Omega \;N_{\text{A}}}+2rh\left\{\gamma_{\text{SL,ZB}\left(\text{WZ}\right)}+\zeta \gamma_{\text{SV,ZB}\left(\text{WZ}\right)}-\gamma_{\text{LV}}\sin\beta \right\}+\frac{\sqrt{3r^{2}}}{2}\gamma_{\text{SN}}$$

where Ω is the solid volume of the Ga-As pair (4.51 × 10^−29^ m^3^), *h* is the height of crystal nucleus, *β* is the contact angle of the droplet, *γ*_LV_ (*γ*_SN_) is the interface energy between the droplet and the vapor phase (between the crystal nucleus and the NW top surface), and *γ*_SL,ZB(WZ)_ and *γ*_SV,ZB(WZ)_ are the interface energies of the GaAs ZB (WZ) crystal nucleus top and side surfaces. Obviously, *h* is the (111) GaAs lattice spacing, which is 0.32639 nm. *γ*_SV,ZB_ and *γ*_SV,WZ_ are the surface energies of (1-10)GaAs and (11-20)GaAs, which are 0.62 and 0.54 J/m^2^, respectively [15]. The interface energy *γ*_LV_ depends on temperature, and we use the following relation: *γ*_LV_ = 0.708 − 0.66 × 10^− 4^ × (*T* − 303) (J/m^2^) [16]. If the crystal nucleus is ZB, *γ*_SN_ = 0. On the other hand, in the case of the WZ crystal nucleus, *γ*_SN_ is 0.023 (J/m^2^), which is half of the GaAs stacking fault energy [17]. We assumed that the contact angle *β* is 45° [18]. Since the NW side surface has a certain asperity, we adopted the parameter *ζ* (0 <*ζ* < 1). Therefore, the maximum *ΔG*_ZB(WZ)_ * and the critical nucleus of Equation 4 for *r* are obtained as follows:

(6) $$\Delta G_{\text{ZB}\left(\text{WZ}\right)}^{*}=\frac{2h^{2}{\left\{\gamma_{\text{SL,ZB}\left(\text{WZ}\right)}+\zeta \gamma_{\text{SV,ZB}\left(\text{WZ}\right)}-\gamma_{\text{LV}}\sin\beta \right\}}^{2}}{\sqrt{3}\left(\frac{h\Delta \mu }{\Omega N_{\text{A}}}\gamma_{\text{SN}}\right)}$$

(7) $$r_{\text{ZB}\left(\text{WZ}\right)}^{*}=\frac{2h\left\{\gamma_{\text{SL,ZB}\left(\text{WZ}\right)}+\zeta \gamma_{\text{SV,ZB}\left(\text{WZ}\right)}-\gamma_{\text{LV}}\sin\beta \right\}}{\sqrt{3}\left(\frac{h\Delta \mu }{\Omega N_{\text{A}}}-\gamma_{\text{SN}}\right)}$$

At steady state, it is known that the probability of crystal nucleation *J*_ZB(WZ)_ is proportional to the S_ZB(WZ)_ region, where a crystal nucleus can form, and Zeldovich factor Z_ZB(WZ)_[19]. The relation is given by

(8) $$J_{\text{ZB}\left(\text{WZ}\right)}\propto Z_{\text{ZB}\left(\text{WZ}\right)}S_{\text{ZB}\left(\text{WZ}\right)}\exp\left(-\frac{\Delta G_{\text{ZB}\left(\text{WZ}\right)}^{*}}{kT}\right)$$

where *k* is the Boltzmann constant, $S_{\text{ZB}\left(\text{WZ}\right)}=3\sqrt{3}{\left\{r_{\text{ZB}\left(\text{WZ}\right)}^{*}\right\}}^{2}$, and the Zeldovich factor is

(9) $$Z_{\text{ZB}\left(\text{WZ}\right)}={\left\{\sqrt{3}\left(\frac{h\Delta \mu }{\Omega N_{\text{A}}}-\gamma_{\text{SN}}\right)/2\pi kT\right\}}^{1/2}$$

Therefore, the probability of twin-crystal nucleation *P* is

(10) $$P=\frac{J_{\text{WZ}}}{J_{\text{ZB}}+J_{\text{WZ}}}=\frac{Z_{\text{WZ}}S_{\text{WZ}}\exp\left(-\frac{\Delta G_{\text{WZ}}^{*}}{kT}\right)}{Z_{\text{ZB}}S_{\text{ZB}}\exp\left(-\frac{\Delta G_{\text{ZB}}^{*}}{kT}\right)+Z_{\text{WZ}}S_{\text{WZ}}\exp\left(-\frac{\Delta G_{\text{WZ}}^{*}}{kT}\right)}$$

**Figure 5:**
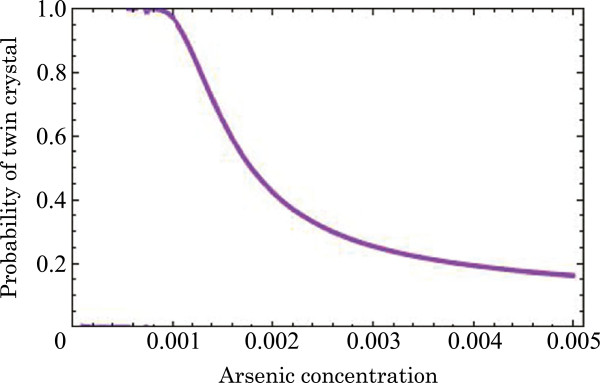
**Relation between the probability of the twin-crystal nucleus and arsenic concentration at 580°C (*ζ*= 0.8).**

This equation indicates that the probability depends on the growth temperature and arsenic concentration. When we assume that *ζ* is 0.8 at 580°C, the relation between the probability *P* and arsenic concentration *c*_As_ is shown in Figure 5. As the droplet arsenic concentration increases with increasing arsenic flux, the probability of twin-crystal nucleation decreases. This phenomenon agrees with the experimental results.

By using the probability of twin-crystal nucleation in Figure 2, we can calculate the arsenic concentration and supersaturation per atom. When the arsenic fluxes are 7.0 × 10^−6^ and 1.9 × 10^−5^ Torr, the arsenic concentrations are 0.11% and 0.38%, and the supersaturations per atom are 53 and 143 meV, respectively. These supersaturation values are smaller than those of Au-catalyzed GaAs NWs (230 to 1,570 meV) [20]. This difference might be due to the difference in the side facet surface. Glas et al. adopted the {111} and {1-100} facets in the ZB and WZ structures in their calculations, respectively. In addition, there might be a difference in the diffusion length between gold and gallium droplets. From the obtained arsenic concentration, we estimate the critical nucleus. When the arsenic fluxes are 7.0 × 10^−6^ and 1.9 × 10^−5^ Torr, the critical nuclei of ZB (WZ) are 1.1 (0.3) and 0.4 (0.1) nm, respectively. This means that the increase of arsenic flux decreases the critical nucleus and increases the growth rate. In the case of high arsenic flux, the size difference between critical ZB and WZ nuclei is small compared with the case of low arsenic flux. This means that the ZB structure appears easily as the arsenic flux increases. Therefore, we could improve the comprehension of the growth mechanism in the self-catalyzed GaAs NWs. This comprehension might support a technological feasibility of a novel device like twin-plane 1D superlattices [21].

## Conclusions

Self-catalyzed GaAs NWs were grown on a (111)Si substrate by MBE-VLS method under various arsenic fluxes. From the TEM observations, we found that the segment size between the twin boundaries depends on the arsenic flux. In order to understand this phenomenon, we attempted to calculate the degree of supersaturation and estimate the probability of twin-boundary formation. When the supersaturation increased with increasing arsenic flux, the size difference between the critical ZB and WZ nuclei decreased. As a result, the ZB structures were easier to obtain as the arsenic flux increased. This qualitatively explained the experimental results and the high probability of the incorporation of twin boundaries.

## Abbreviations

GaAs: gallium arsenide; MBE: molecular beam epitaxy; NW: nanowires; Si: silicon; TEM: transmission electron microscope; VLS: vapor-liquid-solid; WZ: wurtzite; ZB: zinc blende.

## Competing interests

The authors declare that they have no competing interests.

## Authors’ contributions

MY conceived, designed, and coordinated the study and drafted the manuscript. JP carried out the experiments and calculations and helped draft the manuscript. HA participated in the coordination of the study. All authors read and approved the final manuscript.

## Authors’ information

MY is an associate professor, JP is a graduate school student, and HA is a professor at the Department of Electrical Engineering and Computer Science, Nagoya University.
